# Ambulance deployment without transport: a retrospective difference analysis for the description of emergency interventions without patient transport in Bavaria

**DOI:** 10.1186/s13049-023-01159-w

**Published:** 2023-12-06

**Authors:** Florian Dax, Heiko Trentzsch, Marc Lazarovici, Kathrin Hegenberg, Katharina Kneißl, Florian Hoffmann, Stephan Prückner

**Affiliations:** 1 Institut Für Notfallmedizin und Medizinmanagement (INM), University Hospital Munich, LMU Munich, Schillerstr. 53, 80336 Munich, Germany; 2 Dr. Von Haunersches Kinderspital, Paediatric Clinic and Polyclinic, University Hospital Munich, LMU Munich, Lindwurmstr. 4, 80337 Munich, Germany

**Keywords:** Patient transport, Integrated dispatch centre, Ambulance service, Emergency call, Reason for call, Prehospital emergency care, Quality management

## Abstract

**Background:**

Not all patients who call the ambulance service are subsequently transported to hospital. In 2018, a quarter of deployments of an emergency ambulance in Bavaria were not followed by patient transport. This study describes factors that influence patient transport rates.

**Method:**

This is a retrospective cross-sectional study based on data from all Integrated Dispatch Centres of the Free State of Bavaria in 2018. Included were ambulance deployments without emergency physician involvement, which were subdivided into ambulance deployments without transport and ambulance deployments with transport. The proportion of transported patients were determined for the primary reasons for deployment and for the different community types. On-scene time was compared for calls with and without patient transport. Differences were tested for statistical significance using Chi^2^ tests and the odds ratio was calculated to determine differences between groups.

**Results:**

Of 510,145 deployments, 147,621 (28.9%) could be classified as ambulance deployments without transport and 362,524 (71.1%) as ambulance deployments with transport.The lowest proportion of patients transported was found for activations where the fire brigade was involved (“fire alarm system” 0.6%, “fire with emergency medical services” 5.4%) and “personal emergency response system active alarm” (18.6%). The highest transport rates were observed for emergencies involving “childbirth/delivery” (96.9%) and “trauma” (83.2%). A lower proportion of patients is transported in large cities as compared to smaller cities or rural communities; in large cities, the odds ratio for emergencies without transport is 2.02 [95% confidence interval 1.98–2.06] referenced to rural communites. The median on-scene time for emergencies without transport was 20.8 min (n = 141,052) as compared to 16.5 min for emergencies with transport (n = 362,524). The shortest on-scene times for emergencies without transport were identified for activations related to “fire alarm system” (9.0 min) and “personal emergency response system active alarm” (10.6 min).

**Conclusion:**

This study indicates that the proportion of patients transported depends on the reason for deployment and whether the emergency location is urban or rural. Particularly low transport rates are found if an ambulance was dispatched in connection with a fire department operation or a personal emergency medical alert button was activated. The on-scene-time of the rescue vehicle is increased for deployments without transport. The study could not provide a rationale for this and further research is needed.

*Trial registration* This paper is part of the study “Rettungswageneinsatz ohne Transport” [“Ambulance deployment without transport”] (RoT), which was registered in the German Register of Clinical Studies under the number DRKS00017758.

## Introduction

The pre-hospital ground-based ambulance service in Bavaria is ensured by the provision of 506 ambulances (during the day), 364 ambulances (at night) and 229 emergency physician locations [9]. The number of ambulance journeys almost doubled between 1994 and 2013 [33]; in the last 10 years, an increase in the number of ambulance deployments has been observed in both urban and rural regions in Bavaria [16]. Emergency deployments without the involvement of an emergency physician increased by 73% from 368,500 to 638,900 between 2010 and 2019 [9]. In 2018, 24% of all emergencies did not involve patient transport [9]. A resulting proportion of transported patients of only 76% must be questioned from both a resource-oriented and an economic perspective, as high professional demands are placed on the crews and equipment of ambulances in Bavaria. This proportion corresponds with the research results of existing studies, which describe that in up to 30% of ambulance deployments there is no patient transport [14, 24, 34]. As a result, patients are served with a highly specialised resource that might not be appropriate for the occurred event. The aim of the present study is to describe deployments that are particularly likely not to involve transport.

Accordingly, the research question is as follows: Do certain reasons for deployment lead more frequently to emergencies without transport deployments, are there differences between urban and rural areas and do the deployment durations of emergencies with and without transport differ?

Therefore, the aim of the study is to create a basis for determining factors, which influence the transport rate in emergency incidents (without emergency physician involvement), allowing an optimisation of human and material resource planning, along with resource weighting and allocation in the future.

## Materials and methods

### Setting

The analyses of this retrospective cross-sectional study are based on rescue service data of the 26 Integrated Dispatch Centres of the Free State of Bavaria. The Integrated Dispatch Centres (Integrierte Leitstellen) can be reached nationwide under the European emergency number 112 and coordinate all emergency rescue operations. Emergency rescue in Bavaria is organised as a two-tier system. Emergency missions are handled by an ambulance, manned by at least one paramedic or emergency paramedic. In addition, a ground or air ambulance with a physician is dispatched for emergency medical services. First responder units that can optionally be dispatched in advance where available. Dispatchers hold a qualification as paramedic or firefighter, and receive further dispatch training [8]. Dispatchers base their decisions on a non-standardised, keyword-based dispatch protocol. Irrespective of the operator, the dispatch centres in Bavaria use a uniform software [31]. Status messages (1 = waiting for mission, 3 = mission taken over, 4 = arrival at scene, 7 = left scene with patient, 8 = arrival at destination (e.g. hospital) are transmitted from the ambulance to the dispatch centres.

### Data source and sample

The “Institut für Notfallmedizin und Medizinmanagement” has been commissioned by the Bavarian State Ministry of the Interior, for Sport and Integration to process and review the Bavarian rescue service data [5]. On this legal basis, the institute receives all data of rescue service deployments from all Bavarian dispatch centres on a monthly basis in anonymised and standardised form. The data records contain information on the reason for the call and time stamps. For the present quantitative analysis, the data transmitted to the Institut für Notfallmedizin und Medizinmanagement from the Bavaria dispatch centers from 01 January 2018 to 31 December 2018 were used.

We included emergencies without dispatch of a physician, where we assumed that the rescue vehicle reached the scene. Deployments were excluded, when the mission was aborted before reaching the scene of the emergency. In cases where less than two status message were submitted, we assumed that the emergency vehicle did not reach the emergency, was withdrawn, or on a test mission. In addition, mission copies for inter-area missions were excluded. We also excluded missions that seemed unplausible (e.g. hospital was documented, but no status message about the patient admission and discharge), and missions where it was not evident whether a transport had taken place or not.

**Fig. 1:**
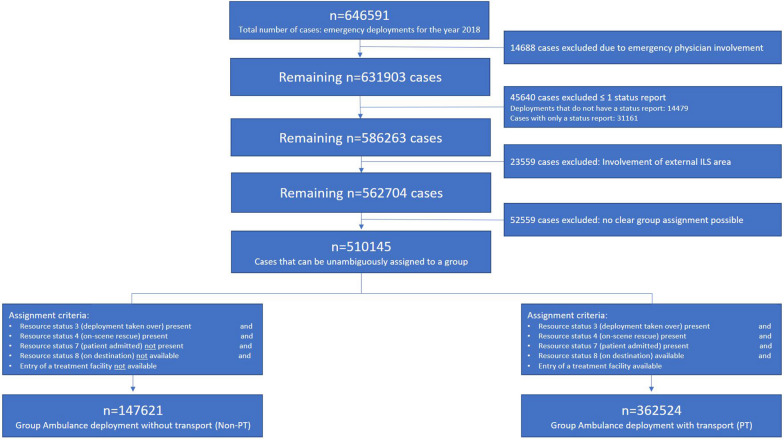
Flow chart of inclusion criteria as well as group assignment of the two groups of emergency events studied

All included deployments were assigned to one of the two groups “emergency mission without transport (non-PT) or “emergency mission with transport (PT)” (see Fig. 1). In some cases dispatchers did document the transport information in data fields not intended for this purpose. These records were manually assigned.

The reason of deployment is standardised from a total of 374 dispatch keywords which are originally specified by the Bavarian state [7] (see Appendix 1).

### Variables and analysis

#### Patient transport rate and reason for deployment

The available reasons for deployment were considered according to the frequency of occurrence in the non-transport vs the transport group. For each reason for deployment, the number of transports was set in relation to the total number of deployments. The odds of a particular reason for deployment being associated with non-transport was indicated by means of an odds ratio and referenced to the category “trauma”.

#### Location of occurrence

Deployment locations were assigned to different community types according to the definition of the Federal Institute for Research on Building, Urban Affairs and Spatial Development (Bundesinstitut für Bau-, Stadt- und Raumforschung): Rural community: < 5000 inhabitants; Small town 5000 to < 10,000 inhabitants; Larger small town 10,000 to < 20,000 inhabitants; Medium-sized town 20,000 to < 100,000 inhabitants; Large town: 100,000 inhabitants or more [11]. The population figures for Bavaria were taken from the official statistical information system of the Bavarian State Office for Statistics (GENESIS online database) [4]. To gain a corresponding insight into whether the deployment figures in urban areas differ from those in rural regions, the two groups were presented in a cross table. Subsequently, the distribution was checked using a Chi^2^ test with calculation of the effect strength by means of the correlation analysis Cramer's V. The odds of a particular community type being associated with non-transport was indicated by means of an odds ratio and referenced to rural communites, since most people in Bavaria live in a rural community [4]. In order to generate comparability with other areas, benchmarking was carried out as it is used in business administration for competitor analyses [29]. For this purpose, the values of the transport and non-transport group per 1000 inhabitants were plotted on the corresponding municipality type. In a multivariate analysis, conspicuous features of the reasons for deployment were presented in relation to the municipality types of the deployment location. For this purpose, the individual reasons for deployment per 1000 inhabitants were analysed in the respective municipality types of the deployment locations according to the transport and non-transport group.

#### On-scene time

Boxplots were created to compare the on-scene times of emergencies with and without transport. The respective intervals were defined as follows:

- On-scene time for emergencies without transport: duration between status 4 (arrival at scene) and status 1 (waiting for mission).
- On-scene time for emergencies with trasport: duration between status 4 (arrival at scene) and status 7 (left scene with patient).
- The on-scene time was limited to a maximum of 80 min in the graphical representation of the boxplot. The statistical calculations included all deployments regardless of the on-scene time. A median and percentile calculation was carried out, the transport and non-transport groups were compared using Mann–Whitney U test and Cramer's V.
- Data analysis took place using both Microsoft Excel 2016 software and IBM SPSS Statistics 25 and R-4.0.3/RStudio 1.3 software.

In order to put the results of the study into context, expert interviews were conducted with one neighbouring dispatch centre (Tyrol, Austria), the body responsible for quality assurance in the Baden-Württemberg rescue service (SQRBW) and an association of dispatch Centers in Germany (Fachverband Leitstellen e.V.) following the analysis.

## Results

A total of 510,145 missions was included. Of these, 147,621 fell into the non-transport-group (28.9%) and 362,524 into the transport group (71.1%).

### Patient transport rate/reason for deployment

The highest proportion of patients was transported due to “birth/delivery” (96.9%), “trauma” (83.2%) and pain (82.9%). The lowest rates were found for emergencies triggered by a fire alarm system (0.6%), emergencies in cooperation with the fire department (“fire with EMS—with and without life-threatening situation”) (5.4%) and emergencies triggered by personal medical emergency alert buttons (18.6%). The reasons for deployment with lowest transport rates showed correspondingly higher odds ratios for dispatch without patient transport when compared to dispatch for “trauma” (see Table 1).

**Table 1:** Patient transport rates of the deployment events

Reason for deployment	Frequency patient transport	Frequency non-patient transport	Frequency Total	Transport rate	OR [95% CI] Reason for deployment (reference: trauma)
Fire alarm system	49	8030	8079	0.6%	810.5 [611.8–1073.7]
Fire with EMS—with and without life-threatening situation	353	6154	6507	5.4%	86.2 [77.4–96.1]
personal emergency response system active alarm	1510	6592	8102	18.6%	21.6 [20.4–22.9]
Technical assistance with emergency medical service	2414	6995	9409	25.7%	14.3 [13.7–15.0]
Child—(up to 12 years) sick	4290	4203	8493	50.5%	4.8 [4.6–5.1]
Intoxication	11,639	9110	20,749	56.1%	3.9 [3.8–4.0]
Aggravation	3223	2353	5576	57.8%	3.6 [3.4–3.8]
Psych	2004	1427	3431	58.4%	3.5 [3.3–3.8]
Consciousness	12,210	7126	19,336	63.1%	2.9 [2.8–3.0]
Child—(up to 12 years) trauma	7110	3559	10,669	66.6%	2.5 [2.4–2.6]
Traffic accident only emergency medical service	17,343	7235	24,578	70.6%	2.1 [2.0–2.1]
Other event/condition	36,925	15,082	52,007	71.0%	2.0 [2.0–2.1]
Heart/circulation	70,764	27,599	98,363	71.9%	1.9 [1.9–2.0]
Respiration	22,779	7321	30,100	75.7%	1.6 [1.5–1.6]
Neuro	32,476	6834	39,310	82.6%	1.0 [1.0–1.1]
Pain	27,723	5712	33,435	82.9%	1.0 [1.0–1.1]
Trauma	105,865	21,406	127,271	83.2%	–
Birth/delivery	3795	122	3917	96.9%	0.2 [0.1–0.2]
Total	362,472	146,860	509,332*	–	–

95% CI = 95% Confidence interval

*In this presentation, only reasons for deployment are taken into account that have more than 1000 deployment events with emergency medical service involvement

### Location of emergency

The analysis of the emergency location showed that a lower proportion of patients was transported in large cities compared to smaller cities or rural municipalities (see Table 2). The Chi^2^ test showed a statistically significant correlation (*p* =  < 0.001) of the variables examined with a low effect size (Cramer’s V 0.127 for 510,145 cases). For the municipality type “Large city”, an increased odds ratio for non-transport of 2.0 [2.0–2.1] was found when referenced to the rural municipalities; the odds ratios of the other municipality types can be taken from Table 2.

**Table 2:** Patient transport frequency according to municipality types for the place of deployment

Municipality type for the place of deployment	Frequency PT	Frequency Non-PT	Total	% Percentage without transport	OR [95% CI] no transport Municipality type for the place of deployment x/ Rural municipality
Large city	107,218	62,761	169,979	36.92	2.0 [2.0–2.1]
Medium-sized city	76,372	27,539	103,911	26.5	1.2 [1.2–1.3]
Large town	58,962	20,090	79,052	25.41	1.2 [1.1–1.2]
Small town	58,069	19,262	77,331	24.91	1.1 [1.1–1.2]
Rural municipality	61,903	17,969	79,872	22.50	–
Total	362,524	147,621	510,145	(28.94)	

95% CI = 95% Confidence interval

**Fig. 2:**
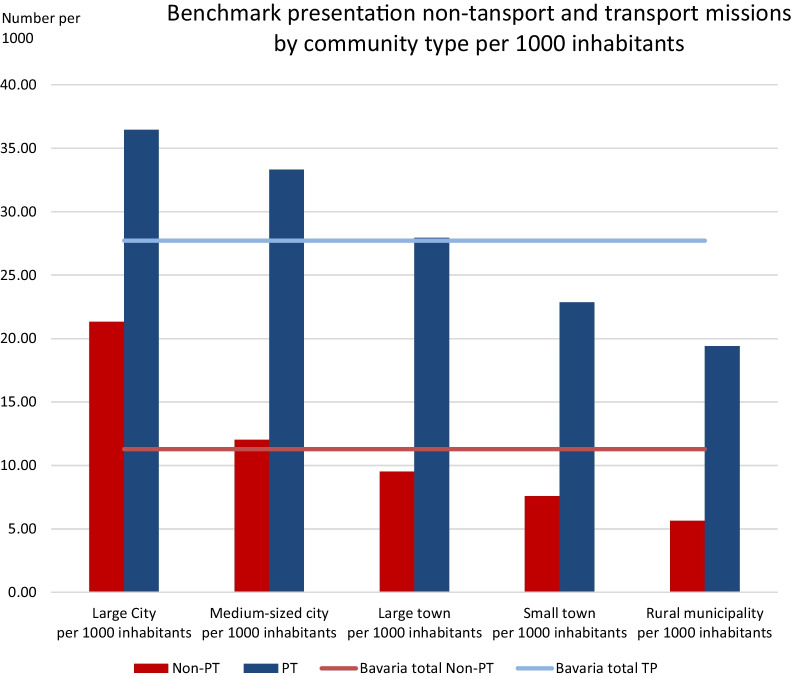
Benchmark presentation of deployments without transport (non-PT) and deployments with transport (PT) by municipality type for the deployment location per 1,000 inhabitants

The type of municipality has an impact on the patient transport rate, being higher in rural areas than in large cities. A Bavarian-wide benchmarking of emergency locations was carried out based on 1000 inhabitants per community type. The benchmark for emergencies without transport is 11.3 deployments per 1000 inhabitants (large city: 21.3 deployments per 1000 inhabitants, rural community 5.6 deployments per 1000 inhabitants), whereas it is 27.7 deployments per 1000 inhabitants for deployments with transport (large city: 36.4 deployments per 1000 inhabitants, rural community 19.4 deployments per 1000 inhabitants). The values of other municipality types can be taken from Fig. 2. Table 3 shows the results of the analysis of the reasons for deployment in relation to the municipality types of the emergency location. Except for “fire alarm system” and “personal emergency response system active alarm”, all other reasons for deployment are most frequently recorded in the municipality type “Large city”.

**Table 3:** Percentage representation of deployments without transport per 1000 inhabitants in the respective municipality types for the deployment locations

Reason for deployment	Large city	Medium-sized city	Larger small town	Small town	Rural municipality
Fire alarm system	22.7	35.7	19.1	13.7	8.8
Fire with EMS—with and without life-threatening situation	22.1	21.9	20.1	18.0	17.8
personal emergency response system active alarm	20.1	31.0	18.4	17.5	12.9
Technical assistance with emergency medical service	45.1	22.8	15.8	9.8	6.5
Child—(up to 12 years) sick	32.7	19.5	20.8	14.5	12.5
Intoxication	57.9	17.9	11.1	8.2	4.9
Aggravation	37.0	29.6	14.4	11.8	7.2
Psych	30.2	22.3	19.5	16.4	11.6
Consciousness	57.9	15.0	12.4	8.4	6.4
Child—(up to 12 years) trauma	33.6	18.3	19.6	15.9	12.6
Traffic accident only emergency medical service	27.5	19.6	18.7	17.2	17.0
Other event/condition	33.6	23.2	18.3	14.1	10.9
Heart/circulation	40.1	19.2	16.5	14.6	9.7
Respiration	39.4	21.1	16.3	13.1	10.2
Neuro	41.7	18.8	16.7	13.2	9.5
Pain	44.3	18.1	16.8	11.6	9.2
Trauma	41.1	19.5	17.3	13.1	9.0
Birth/delivery	65.2	10.7	16.9	4.4	2.8

#### On-scene time

Of 510,145 records, on-scene time was missing fot 5659 records, so 503,576 (141,052 non-transport and 362,524 transport) records were included in the calculation.

**Fig. 3:**
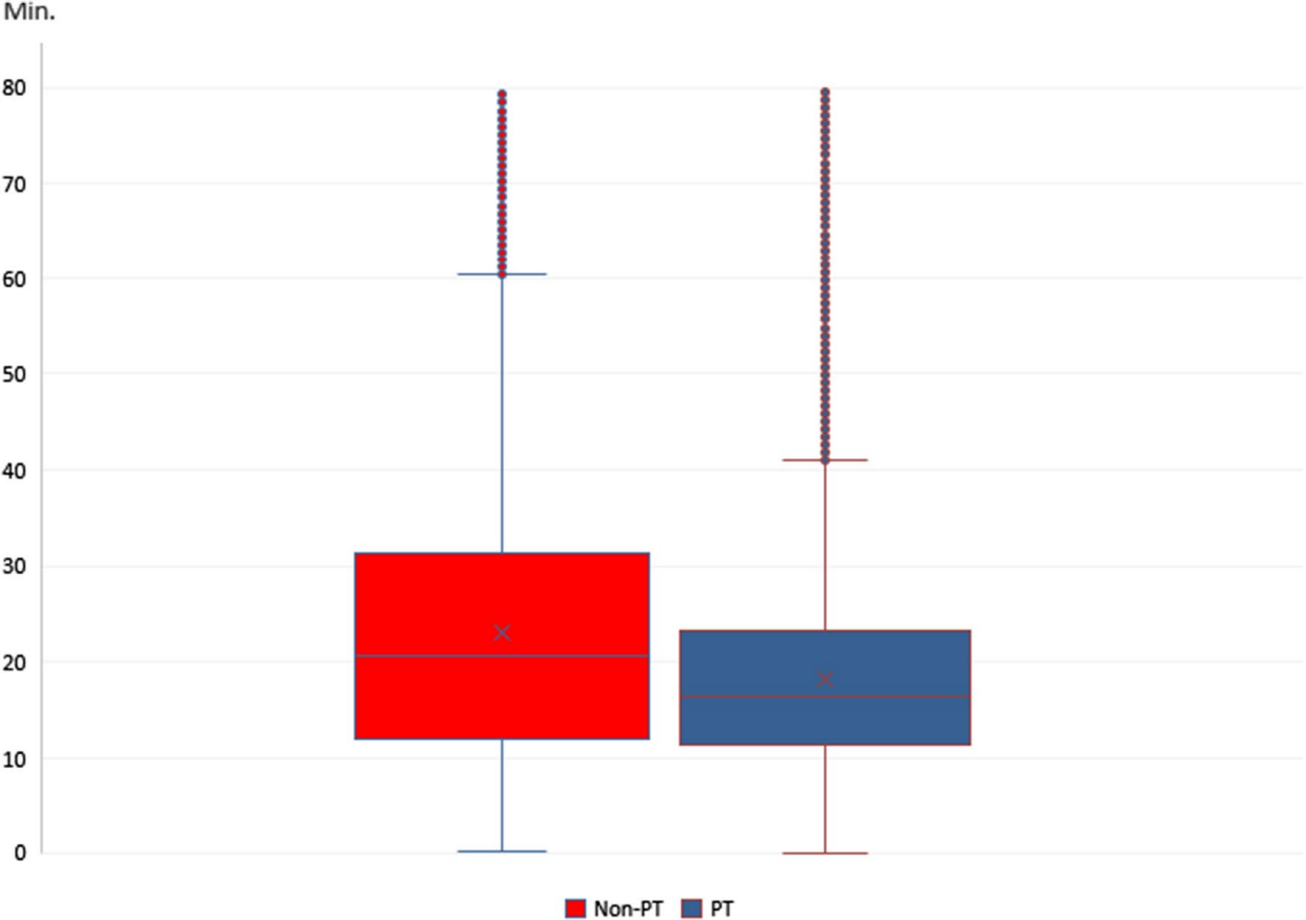
On-scene time of non-PT and PT deployments

The median difference between emergencies with and without transport is 4.29 min (see Fig. 3): median on-scene time for deployments without transport was 20.77 min (n = 141,052) and for transported patients was 16.48 min (n = 362,524). The high time differences in the 95th percentile were striking (see Table 4). A Mann–Whitney U test showed significant differences (*p* =  < 0.001) between the two groups, with a medium effect size (Cramer's V = 0.14, Z = 102.0, n = 503,576).

**Table 4:** List of on-scene times in minutes and by reason for deployment

Reason for deployment	Non-PT median (Q1-Q3)	PT median (Q1-Q3)
Fire alarm system	9.02 (5.33–13.78)	21.58 (13.63–31.56)
Fire with EMS—with and without life-threatening situation	16.20 (8.72–29.75)	24.37 (18.14–34.45)
personal emergency response system active alarm	10.55 (6.60–17.62)	23.82 (17.67–31.24)
Technical assistance (THL) with emergency medical service	17.17 (9.95–28.13)	32.34 (23.53–42.60)
Child—(up to 12 years) sick	22.30 (15.17–30.62)	14.93 (10.82–20.37)
Intoxication	16.27 (9.17–26.85)	15.05 (10.17–22.03)
Aggravation	15.17 (8.41–24.60)	14.97 (9.48–22.62)
Psych	26.60 (16.08–39.92)	18.48 (11.95–28.13)
Consciousness	25.30 (15.65–36.22)	19.88 (14.20–26.97)
Child—(up to 12 years) trauma	18.80 (12.83–26.22)	12.43 (9.03–16.97)
Traffic accident only emergency medical service	17.26 (9.68–26.55)	16.23 (11.52–22.70)
Other event/condition	20.92 (12.65–31.63)	15.60 (10.83–22.25)
Heart/circulation	26.82 (18.73–36.63)	18.53 (13.25–25.12)
Respiration	27.70 (19.44–38.13)	18.97 (13.68–25.90)
Neuro	28.33 (18.92–39.68)	19.20 (13.93–25.86)
Pain	24.28 (16.57–33.93)	14.55 (10.18–20.55)
Trauma	19.42 (12.40–29.37)	14.80 (10.18–20.93)
Birth/delivery	19.07 (12.45–31.45)	10.25 (7.12–15.03)
Median total	20.77	16.48
N	141,051	362,277

Q1 = first quartile/lower quartile, Q3 = third quartile/upper quartile | reasons for deployment according to appendix

To identify, whether individual deployment reasons explain the differening on-scene times, the corresponding times were presented according to the different deployment reasons for emergencies with and without transport. Significantly lower on-scene times can be seen for the reasons “fire alarm system” and “personal emergency response system active alarm” for ambulance deployments without patient transport (Table 4). For transports, low on-scene times are recorded for the reason “birth/delivery”.

## Discussion

The proportion of patients transported was related to the reason for dispatch as well as to whether the emergency location was rather rural or urban. Particularly low transport rates are found for dispatch in association with the fire brigade and in connection with personal, automatic medical emergency response systems, as well as deployments in urban areas. In addition, longer on-scene times were identified for emergencies without patient transport compared to emergencies with subsequent transport.

### Incidence of non-transport

A study by Infinger, Studnek et al. [19] showed that for an ambulance service with an average annual call volume of about 90,000 calls, for two patients per day, the correct resource would be a nurse consultation and not the dispatch of an ambulance. A retrospective cohort study from Finland by Hoikka, Silfvast et al. [18] concluded that in 13,354 ambulance calls, 41.7% of patients were not transported. However, the comparability with the present study is limited, as Hoikka, Silfvast et al. also included emergency physician deployments and therefore includes physician decisions not to transport. In our approach, we deliberately chose to leave out these medical decision, as our primary aim was to shed light on the dispatching decisions. Khorram-Manesh, Lennquist Montán et al. [21] demonstrated a discrepancy between the dispatch centre assessment and the actual priority, resulting in unnecessary hospital transports. Jensen, Carter et al. present a variety of evaluation methods of dispatching alternatives but conclude that comparability is difficult to establish due to the heterogeneity of the systems [20]. Discussing our findings with experts from a neighbouring dispatch centre, a professional association and quality assurance body revealed that both within Germany and in the neighbouring Austrian state of Tyrol, comparisons of ambulance dispatch without patient transport are complicated by differing legal bases and billing procedures. This indicates that, within Germany, there is a need for a common database for emergency service data (analogous to the German Resuscitation Register [15, 22] or the German Trauma Register DGU® [17, 35]) as a basis for future research. Even though there is a standardized dataset (minimaler Notfalldatensatz MIND), which contains a defined set of characteristics that are required for the documentation of prehospital emergency medical services [13, 25] and is authorised by the Deutsche Interdisziplinäre Vereinigung für Intensiv- und Notfallmedizin [German Interdisciplinary Association for Intensive Care and Emergency Medicine] (DIVI), it is apparently not implemented in a uniform manner.

### Reasons for emergency missions without transport

Some emergencies without transport could essentially be non-emergency deployments. However, other reasons can also be responsible for why a patient is not taken to a treatment facility. Billittier, Moscati et al. already described in 1996 that, in addition to medical reasons for patients, the lack of alternative transport options also plays a role in the use of the emergency medical services [10]. Laukkanen et al. [23] researched that ambulance personnel are usually able to safely assess patients at the scene when there is no patient transport.

Low transport rates could possibly be due to the lack of adequate deployment of resources. In view of the very low transport rates for the reason “fire alarm system”, might be necessary to rethink whether it is appropriate to automatically dispatch ambulances when planning the fire alarm system. Dispatch due to personal emergency response system alarm buttons going off could involve cases where the personal emergency response system call centres do not have enough resources for their own transport service. This circumstance could be improved by the obligatory introduction of an on-call driving service for personal emergency response system call centres, because their own resources could take over these deployments instead of an ambulance.Another factor influencing the transport rate is the community type for the emergency site: in urban areas, the proportion of patients transported is lower than in rural areas, although there are more publicly accessible care services such as on-call practices or day clinics. This partial result of our study corresponds with other studies, such as the analysis of the performance level in the rescue service for the years 2016 and 2017 by the Federal Highway Research Institute (“BAST Study”). In this study, the distribution of false trips in rural regions is 2.2% and in urban regions 8.9% [30]. Possible reasons for this difference may be the different composition of the patient collective, e.g. with regard to socio-economic characteristics or the anonymity of big cities. The differences in the transport rate for the reason intoxication (per 1000 inhabitants: 57.9% without patient transport in the large city vs. 4.9% without patient transport in the rural community) could be indicative of this. Further explanations may be differing patient compliance and better accessibility of specialised clinics in urban areas. The influence of the disposition quality cannot be derived from the available data.

### Duration of missions

Transport by EMS without medical justification would be counter-intuitive to demand-oriented planning of health care, as outlined in the expert opinion of the expert council (Sachverständigenrates zur Begutachtung der Entwicklung im Gesundheitswesen) [27]. The longer duration of missions without transport might be explained by documentation efforts that are usually included in the on-scene time. This does not only inculde the documentation of the actual mission, which may not even be necessary in the case of incorrect deployments, but also the time spent on filling out transport refusal declarations.

In addition, the on-scene time does not indicate when the ambulance will be available again for further deployments—the transport interval (transport and transfer to a treatment facility) must be added to the PT group. However, very low on-scene times for non-transport missions with the reasons “fire alarm system” (9.02 min on-scene time) and “personal emergency response system active alarm” (10.55 min on-scene time) could indicate incorrect deployments for the ambulance service.

### Potential alternative services

It should be questioned whether adding further low-threshold rescue vehicles to the system would make sense, since some patients may not need the full human and technical resource of an ambulance. In some regions of Germany, additions to the emergency medical services are emerging, such as the pilot project of community emergency paramedics in Oldenburg [2, 32] or rescue response vehicles in Schleswig–Holstein [26] and Bavaria [3], which can be alerted additively or as a substitute. With these response resources, patients who do not require transport capacity can be treated on site. In Rhineland and Hamburg, more than half of the emergency outpatients were treated in hospital in 2018. For 55 per cent of them, only the emergency flat rate was billed—an indication that the patients might have been better off in the statutory emergency service [1, 28].

### Limitations

For the interpretation of reason for deployment it must be considered that the documentation of a reason at dispatch might sometimes differ from the evaluation by the crew at scene. Also, in case a dispatcher made changes to the documentation in the course of the mission (e.g., because a triggered fire alarm system turned out to be a real fire event), the altered reason for deployment is analysed. Dispatchers also have some leeway when it comes to choosing the keyword: For example, when a dispatcher receives a message via a personal emergency response system call centre indicating a fall, he or she can decide whether to select choose “personal emergency response system active alarm” or “fall” as reason for dispatch.

The conclusions of this study are based on the data collection of integrated dispatch centres in Bavaria and thus Bavarian legislation and EMS system. A transfer to other settings with differing legal frameworks, EMS vehicles and documentation is difficult. In Bavaria, there are state-wide standardized user fees controlled by the Zentrale Abrechnungsstelle für den Rettungsdienst Bayern GmbH [Central Billing Office for the Bavarian Ambulance Service GmbH] (ZAST)) [6]. Due to this legal framework, there are no monetary incentives for the provider of EMS to transport patients to a care facility every time. This might be different under other framework conditions—nationally and internationally.

The present study does not give any indication as to why a patient was not transported. For this, further investigations are necessary, such as interviews with the ambulance staff or an analysis of the mission documentation.

## Conclusions

This study describes the frequency of emergency deployments of ambulances without patient transport for the Free State of Bavaria during a one-year observation period. (2018) Differing transport rates were found depending on the reason for the dispatch. Deployments without transport were observed particularly frequently for alarms related to fire alarms and personal emergency response system active alarm. Yet for some other reasons less than two thirds of the patients were transported to a hospital. Although ambulances that do not transport are presumably more quickly ready for action again than those that transport, the on-scene time for most reasons for dispatch was longer. This could be due to more time needed for on-scene care, clarification of the situation or also due to the documentation required afterwards. Assuming that not every deployment without transport is synonymous with an erroneous deployment, it can be concluded, that resource management could probably be improved for certain deployment reasons. Since there were differences between urban and rural areas, resource planning could also be adapted to the regional setting accounting for urban and rural infrastructure. Further studies on the disposition quality in different settings (urban/rural) are needed.

## Data Availability

Please contact author for data requests.

## Appendix 1: Reasons for deployment and their explanations

**Table:**

Fire alarm system	Automatically triggered fire or smoke alarm system with dispatch of at least one ambulance
Fire with EMS—with and without life-threatening situation	Fire event with dispatch of at least one ambulance
personal emergency response system active alarm	Forwarding of an emergency call by the personal emergency response system call centre in the event of active alarms of the personal emergency response system (e.g. activation of the emergency call button for senior citizens)
Technical assistance with rescue service	Technical assistance by the fire brigade with dispatch of at least one ambulance (e.g. in case of trapped persons)
Child—(up to 12 years) sick	Acute symptomatology that cannot be referred to the general practitioner or the medical officer, e.g. ingestion of potentially toxic substances without clinical symptoms
Intoxication	Intoxication with potentially toxic substances without evidence of life-threatening disturbance of the heart, circulation or consciousness
Aggravation	Injury after a fight without acute life-threating situation
Psych	Psychiatric condition that does not permit referral to the general practitioner or the emergency medical service
Consciousness	New-onset, non-escalating disturbance of consciousness with exclusion of a life-threatening indication, which cannot be referred to the general practitioner or emergency medical service
Child—(up to 12 years) trauma	Acute injuries requiring prompt care with no indication of vital risk, taking into account the mechanism of the accident
Traffic accident only rescue service	Acute injuries requiring prompt care with no indication of life-threatening danger, taking into account the mechanism of the accident
Other event/condition	Metabolic derailment without clinical symptoms, gastrointestinal or gynaecological haemorrhage, nosebleed, body temperature derailment
Heart/circulation	New-onset, non-escalating cardiac or circulatory symptoms with exclusion of a life-threatening indication, which cannot be referred to the general practitioner or emergency medical service
Respiration	New-onset, non-escalating respiratory symptoms with the exclusion of a life-threatening indication that cannot be referred to the general practitioner or emergency medical service
Neuro	New onset emergency medical services neurological deficits (e.g. stroke symptoms) without disturbance of consciousness, condition following a single seizure, other neurological condition that does not permit referral to the general practitioner or the emergency medical service
Pain	New onset, non-escalating pain that does not allow referral to the general practitioner or the emergency medical service
Trauma	Acute injuries requiring prompt treatment with no indication of life-threatening danger, taking into account the mechanism of the accident, electrical accident without symptoms
Birth/delivery	Contractions, loss of amniotic fluid, birth not imminent

## Abbreviations

DIVI: Deutsche Interdisziplinäre Vereinigung für Intensiv- und Notfallmedizin [*German Interdisciplinary Association for Intensive and Emergency Medicine*]
MIND: Minimaler Notfalldatensatz [*minimum emergency data set*]
PTQ: Patiententransportquote [*patient transport quota*] (proportion of patients transported to a treatment facility out of the total number of emergency calls)
Non-PT: Rettungswageneinsatz ohne Transport [*ambulance deployment without transport*]
SQR-BW: Stelle zur trägerübergreifenden Qualitätssicherung im Rettungsdienst Baden-Württemberg [*Office for Interagency Quality Assurance in the Baden-Württemberg Rescue Service*]
StMI: Bayerisches Staatsministerium des Innern, für Sport und Integration [*Bavarian State Ministry of the Interior, for Sport and Integration*]
PT: Transport (here: ambulance deployment with patient transport)
ZAST: Zentrale Abrechnungsstelle für den Rettungsdienst Bayern GmbH [*Central Settlement Office for the Bavarian Rescue Service GmbH*
